# Preliminary Process and Microstructure Examination of Flux-Cored Wire Arc Additive Manufactured 18Ni-12Co-4Mo-Ti Maraging Steel

**DOI:** 10.3390/ma14216725

**Published:** 2021-11-08

**Authors:** Krzysztof Pańcikiewicz

**Affiliations:** Faculty of Metals Engineering and Industrial Computer Science, AGH University of Science and Technology, A. Mickiewicza 30, 30-059 Kraków, Poland; krzysztof.pancikiewicz@agh.edu.pl

**Keywords:** wire arc additive manufacturing, flux-cored wire, maraging steel, martensite, austenite

## Abstract

The production of large-size elements using additive manufacturing is a constantly evolving field that includes technological and material solutions. There is a need for a detailed analysis of the process and the products thus manufactured. In line with this trend, the flux-cored wire arc additive manufactured process and the part made of 18Ni-12Co-4Mo-Ti maraging steel were examined. The interpass temperature below 150 °C, the variation of the starting point and the gas flow of 12 L/min with a pre-flow of 2 s ensure the correct shape of the layers. The manufactured part underwent chemical composition analysis, macro- and microscopic examination and hardness measurements; in addition thermodynamic calculations were performed. The part is divided into a light-etched area (bottom part of the sample) with a hardness of 375 ± 12 HV10 and a dark-etched area (top part of the sample) with a hardness of 525 ± 11 HV10. Microscopic observations in the last layers showed supersaturated martensite with primary precipitates of μ-phase intermetallic compounds in intercellular spaces. In the earlier layers aging martensite with austenite and primary precipitates of intermetallic compounds were revealed. The share of austenite was 11.435 ± 1.313%.

## 1. Introduction

Wire-arc additive manufacturing (WAAM), initiated in the 1925s by Baker [1] to deposit metal ornaments, is a continuously evolving process of additive manufacturing. WAAM is a direct energy deposition technique that builds up a part in a layer-by-layer fashion, each layer being constituted of interlaced weld layers. Basically, WAAM adopts arc welding tools—power sources, torches, wire feeding system—and wire as feedstock. Movement can be provided by robotic systems or numerically controlled gantries, the most commonly used equipment for gas metal arc welding with solid wire electrode or non-consumable tungsten electrode and plasma arc welding. Continuous development of welding methods, techniques and technologies (CMT, ColdArc etc.) provide a solid basis for the development of this method as well. Controlled dip transfer of metal drops allows to obtain beads with excellent quality, lower thermal heat input and nearly without spatter. WAAM offers lower maintenance costs and a higher deposition rate (up to 7–10 kg/h) than other AM processes [2,3]. Both new ways of conducting the process [4,5,6,7,8,9,10], strategies [11,12], and the use of new filler materials are investigated. WAAM parameters have a significant influence on the geometry and microstructure, which was studied by Dinovitzer et al. [13]. By laying successive layers of appropriate dimensions, it is possible to shape the final form of the product. Unfortunately, a few starts and stops of the arc passing from one layer to another cause transient phenomena that slow down the printing process, which can be eliminated by using CTPP—Continuous Three-dimensional Path Planning [14] or Robot-Centered Path-Planning [15]. Attention is also paid to the detection of defects using High Dynamic Range Images [16]. The use of additional treatments such as active cooling [17], on-line vortex cooling [18], ultrasonic vibration [19] and post heat treatment [5,20] is examined. The influence of interpass temperature/delay [21] or heat input [8,22,23,24,25] is analyzed, in the context of distortion and deformations [26,27].

The fundamental materials used for WAAM are steels, despite significant differences between the grades in their chemical composition, crystal structure and properties. It is possible to use additives on unalloyed steels [28], low-alloy steels [20,29], mild steel [12] and high-strength low-alloy steels [30], yet an unfavourable phase can be obtained in the structure, as reported by Nemani et al. [29] after WAAM, with the use of ER70S filler material. Austenitic stainless steels also qualify as trouble-free materials, which is proved by Xie et al. [9], Yao et al. [22] and Wu et al. [23]. Roy et al. [2] successfully made a part of AISI 410 martensitic stainless steel; similarly, Lervåg et al. [10] for superduplex steel and Tain et al. [31] for 10CrNi3MoV steel.

The WAAM process also uses non-ferrous metals. The inferior weldability of aluminium alloys compared to steel is transferred into the WAAM process. The variable solubility of gases after the transition from liquid to solid, and the crystallization character, determine the potential presence of porosity or microporosity and hot cracks, also transcrystallisation influences the high anisotropy of mechanical properties [6,17,18,32,33,34]. In the case of titanium alloys, the problem is their high reactivity to oxygen and coarse grains, which can be regulated by interlayer rolling/forging or inoculation [3,4,5,7,35,36,37,38]. Copper alloys [19,39], nickel alloys [40,41,42], magnesium alloys [43,44], tantalum [45,46], tungsten [47,48] and high-temperature resistant MoNbTaWTi high-entropy alloy [49] produced in the WAAM process were also subjected to detailed analysis.

A separate group of steels, which is a little-known area in the field of WAAM products, is maraging steels. The name comes from the two terms—“mar” for martensite and “aging”, which indicates that the proper microstructure for these steels is aged martensite. Aging allows for the release of strengthened intermetallic compounds. Xu et al. [50] conducted preliminary WAAM tests using CMT (Cold Metal Transfer) welding technology and 18Ni-8Co-4.5Mo-0.5Ti alloy obtained from solid wire MARVAL 18 S. Various manufacturing strategies were tested, surface quality and lack-of-fusion defects were analyzed. A detailed microscopic analysis [51] revealed heterogeneity of the structure across the cross-section of the part. Transient thermal cycling resulted in partial aging and nonuniform formation of intermetallic compounds along the building direction. At the top of the wall, supersaturated martensite was observed, while at the bottom, aged martensite with retained and reverted austenite. These observations are confirmed by the research of Xiaowei et al. [52]. Xu et al. [53] also studied mechanisms of oxide accumulation and the influence of oxides on subsequent deposition in the 18Ni-8Co-4.5Mo-0.5Ti alloy. It was found that the deoxidizing elements in the wire combine with oxygen to form oxides on the surface of the layer, and the dispersed nanooxides are trapped in the material and increase ultimate tensile strength by 11%.

The purpose of this work was to analyse the possibility of wire arc additive manufacturing with the use of flux-cored wire feedstock and to show the mechanism of structure formation. This goal was achieved by metallographic macro- and microscopic observations, EDS analysis, simulations and hardness measurements. In this study, the WAAM process was carried out with the use of 18Ni-12Co-4Mo-Ti material, richer in Co than the studies by Xu et al. [50,51,53] and carried out with the use of flux-cored wire.

## 2. Materials and Methods

**Figure 1:**
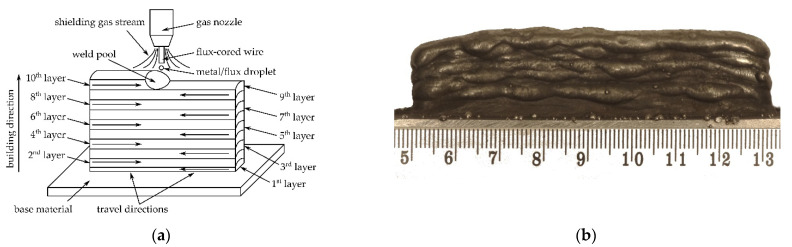
Wire arc additive manufactured 18Ni-12Co-4Mo-Ti maraging steel part: (**a**) manufacturing strategy, (**b**) general view.

For evaluation, a plate with dimensions of 80 mm was used × 20 mm × 8 mm after flux-cored wire arc manufacturing. The manufacturing process was carried out on a robotic MIG / MAG welding station, equipped with a Fanuc ArcMate 100i welding robot (Fanuc, Oshino, Yamanashi, Japan) and a Kemppi ProMIG set - Kemppi PRO4200 welding heat source, Kemppi ProMig 520R controller, Kemppi ProCool 30 cooler and Kemppi ProMig 120R wire feeder (Kemppi Oy, Lahti, Finland). COREWELD NiCoMo metallic flux-cored wire (metal tube with flux wrapped inside) with a diameter of Ø1.2 mm manufactured by Metalweld Fiprom Polska Sp. z o.o. was used for manufacturing as a feedstock. The functions of the flux are protecting the molten metal from the air, reduction of the solidification rate of the metal (facilitating the release of gases and non-metallic inclusions from the liquid metal), regulating the chemical composition and refining metal. The electric arc shield was provided using 99,998% (4.8 type) argon at a flow rate of 12 L/min. The parameters of the additive manufacturing process are shown in Table 1 and the manufacturing strategy with a general view of the part is shown in Figure 1.

The chemical composition of the plate was identified with the Foundry-Master (Worldwide Analytical Systems, Uedem, Nordrhein-Westfalen, Germany) optical emission spectrometer (OES). Observation of the macro- and microstructure was performed with a Leica Stereozoom S9i and Leica DM/LM (Leica, Wetzlar, Germany) light microscope (LM) with a bright field (BF) and a Phenom XL (Thermo Fisher Scientific, Waltham, MA, USA) scanning electron microscope (SEM) with backscattered electrons (BSE). Energy dispersive spectroscopy (EDS) analysis was used to analyse the chemical composition in the microarea. X-rays excited by an electron beam with an accelerating voltage of 20 kV and a current of 10 nA were used for the analysis, similar to SEM-BSE imaging. For microscopic examination, the samples were etched in a 4% alcoholic nitric acid solution. Image analysis was carried out using ImageJ 1.49v software (Wayne Rasband, National Institutes of Health, Bethesda, MA, USA) on 5 images to determine the percentage of austenite in the structure. Hardness measurements were made using the Vickers method with an intended load of 10 kG (98.07N), using a Zwick/Roell ZHU 187,5 (Zwick Roell Group, Ulm, Germany) hardness tester. Thermodynamic analyses were performed by Thermo-Calc 2021a (Thermo-Calc Software AB, Solna, Sweden) with the TC-FE7 database (Thermo-Calc Software AB, Solna, Sweden).

## 3. Results and Discussion

### 3.1. Analysis of Additive Manufacturing Process

The accepted parameters of the WAAM process (Table 1) allowed the production of a part in the form of a wall with an average thickness of 8 mm, determined by the layer width. The use of an interpass temperature below 150 °C ensured a comparable width of the performed layers. WAAM without control of the interpass temperature led to an increase in the volume and surface area of the weld pool due to the deeper remelting of the previous layer. As a consequence, it led to an intensive drip of liquid metal down the walls of the part as a result of exceeding the critical surface tension force, which kept the pool on the surface of the last layer. Kozamernik et al. [54] came to similar conclusions by examining the influence of the interpass temperature on the deviation from the geometry of the planned product, indicating that 150 °C is sufficient to obtain a deviation of less than 2%. Similarly, Knezović et al. [55] indicate 150 °C as appropriate.

Maintaining a constant starting and ending point leads to a wall of different heights along its length, as also observed, e.g., by Voropaev et al. [28]. In the start area, the wall height was lower than in the finish area. The assumption of the strategy presented in Figure 1a allows one to obtain a uniform wall height and ensure greater stability of the WAAM process. This strategy also limits the phenomenon of an increase in the thickness of the privileged crystallites during transcrystallisation.

A shielding gas flow of less than 10 L/min causes global or local porosity, as also observed by Knezović et al. [55], where the groups of pores are on the outer sides of the duplex steel wall. The use of a flow of 12 L/min in combination with slag covering the face of the layer guarantees no porosity, except for the arc ignition point. The use of a gas pre-flow, preceding the ignition of the arc by 2 seconds, reduced the occurrence of porosity at the point of ignition of the arc, observed in previous tests without gas pre-flow.

Regardless of the tested process parameters, a local occurrence of the spatter is observed—single, small droplets falling outside the arc area and falling outside the weld pool. Assuming that the base material will not be a part of the finished product and the surfaces of the manufactured product will be machined, loose spatter, lightly adhering spatter and strongly adhering spatter will not be a problem.

### 3.2. Chemical Composition Analysis

The chemical compositions of the additive manufactured part are presented in Table 2. Based on manufacturer data, the expected steel grade was assigned, corresponding to 1.6356 steel. Significant deviations between the manufacturer data and OES results in carbon, silicon and nickel content were found.

The higher carbon content in the matrix increases the dislocation density and thus strengthens the solid solution, reducing the plasticity of the alloy [56]. Assuming that the WAAM process is the last and no deformation is anticipated, this is not a hindrance. The negative effect of a higher carbon content may be the formation of brittle titanium carbides, especially when they are located at the grain boundaries [57] and molybdenum carbides (Figure 1). Precipitation of carbides will cause a decrease in the amount of intermetallic phases of these elements after subsequent aging. The increased carbon content leads to an increased amount of residual austenite [56,57]. The increased silicon content may be responsible for the formation of the G phase with the composition Ti6Si7Ni16, observed by Sha et al. [58] in the 0.1C-11.2Cr-9.1Ni-1.3Mo-1.2Al-1Ti-1Si model maraging steel after age 520 °C/5 h. The increased content of silicon in the wire in relation to grade 1.6356 is understandable due to the fact that the flux-cored wires must contain deoxidizers, denitrifiers and slag formers (such as an oxide of silicon) to protect the molten weld pool form the atmosphere. Silicon also provides good metal fluidity, so that the feedstock is wetted for complete fusion and produces a smooth, flat layer [59]. The nickel content is lower than declared by the manufacturer, but not much different from the range for steel 1.6356. The lower nickel content, the less intermetallic compounds that can be released during aging. The qualitative Ti addition presented by the manufacturer is small and amounts to 0.332%. A much lower value in relation to the 1.5–2.0% range provided for steel 1.6356 may be due to the burnout of titanium during the process [60].

### 3.3. Structure Examination

**Figure 2:**
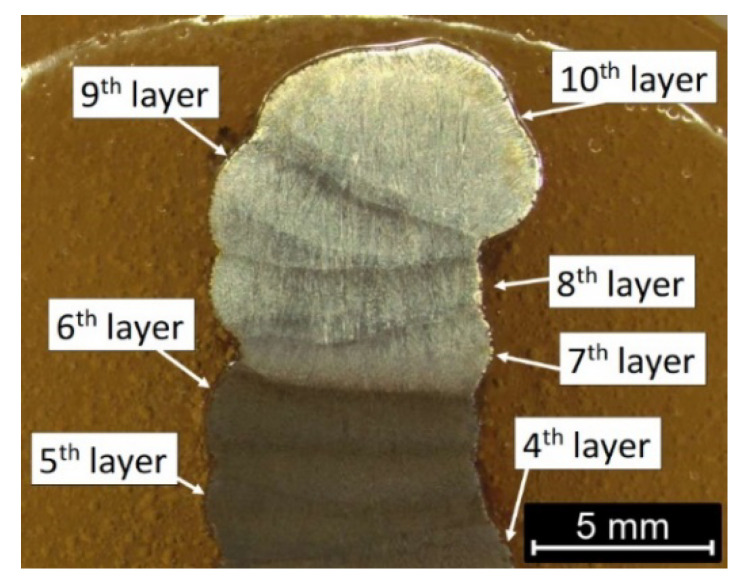
Macrostructure of the wire arc additive manufactured 18Ni-12Co-4Mo-Ti maraging steel part.

Macro- and microstructure analysis revealed the presence of two distinctly different zones—light and dark etching zones—on the cross-section of the wire arc additive manufactured part. Figure 2 shows the overall macrostructure of the part in the area of the last 7 layers. Revealing the structure required a change in the etching technique. Layers 1–6 were exposed during short-term etching, but the remaining layers (7–10) were not etched. Increasing the etching time led to the overpickling of the microstructure of layers 1–6 and the disclosure of the structure in layers 7–10. The effect of uneven etching of different areas in maraging steels is observed, among others, in the heat-affected zone in welded joints [61,62,63,64] and WAAM products [51,52]. Moreover, because the heat is dissipated from top to bottom and is limited in the direction of the wall thickness, it facilitates the formation of a columnar grain, observed in Figure 2. In the region of the 10th layer at a depth of about 0.5 mm from the face surface, a macroscopic solidification grain boundary is visible along the face. This boundary was created as a result of the contact of the crystallization front running from the fusion line with the crystallization front on the outer surface of the layer. The occurrence of an additional crystallization front required the creation of conditions for nucleation on the surface related to thermal supercooling. In the present case, the nucleation site may also be the liquid metal/slag interface. Internal defects are not observed. Unfortunately, the collinearity of the individual layers was not maintained, but this defect, however, does not affect the microstructural analysis. The fusion profile was similar to the “c” type, proposed by Ríos et al. [65]. This effect has been achieved using flux-cored wire, where, unlike the solid wire, the droplets detach from the tubular sheath and thus have a much larger incidence area. This results in a more evenly distributed heat energy of the arc over the surface of the weld pool. The fusion is wider and shallower, with a regular circular shape.

**Figure 3:**
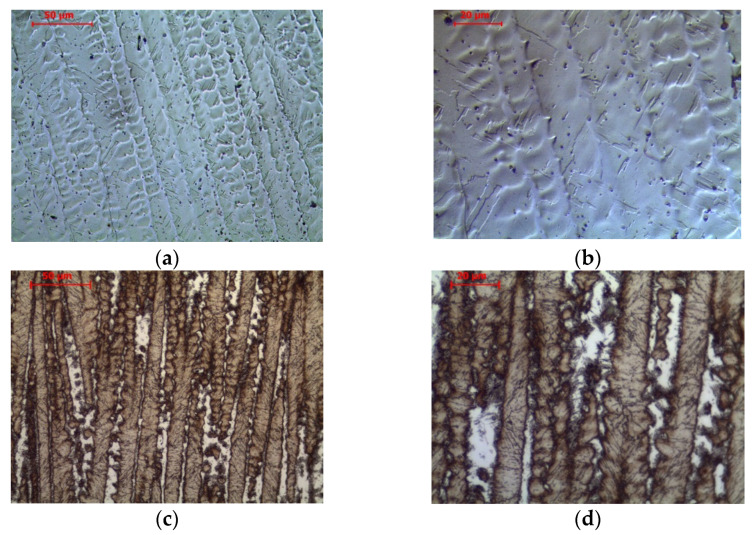
Microstructure of wire arc additive manufactured 18Ni-12Co-4Mo-Ti maraging steel in the: (**a**,**b**) 10th layer, (**c**,**d**) 6th layer. 4% Nital etched.

**Figure 4:**
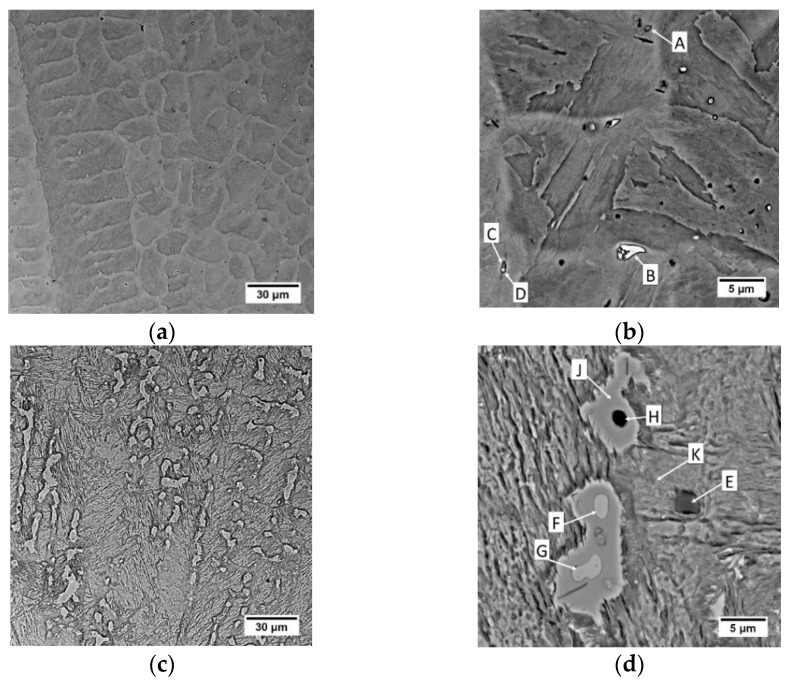
Microstructure of wire arc additive manufactured 18Ni-12Co-4Mo-Ti maraging steel in the: (**a**,**b**) 10th layer, (**c**,**d**) 6th layer. 4% Nital etched.

The etching effects observed on the macrostructure were transferred into microstructural observations. A martensitic structure is observed in the area of the last layers (Figure 3a,b and Figure 4a,b). A similar structure was observed by Viswanathan et al. [66] and Shamantha et al. [67], interpreting it as massive blocks or packets of martensite consisting of bundles of parallel, heavily dislocated laths. The cellular structure of the crystallization was revealed by adjusting the contrast with the aperture diaphragm in LM and the use of a backscattered electron detector in SEM analysis. During non-equilibrium crystallization, as in WAAM, segregation of the chemical composition in the micro-area may occur. Segregation is determined by the solute partition coefficient, which characterizes the redistribution of the solute at the solid-liquid interface. Mn, Si, Ni, Mo and Ti are characterized by the value a solute partition coefficient below 1. This indicates the possibility of segregation of these elements at the cell boundaries in the analyzed maraging steel.

In addition, a large number of primary precipitates is observed in the structure. Their precipitation does not have a positive effect on the strengthening of the steel—they are not finely dispersed, they reduce the content of strengthening elements in the matrix, which later precipitate during aging in the form of fine-dispersion precipitates.

**Figure 5:**
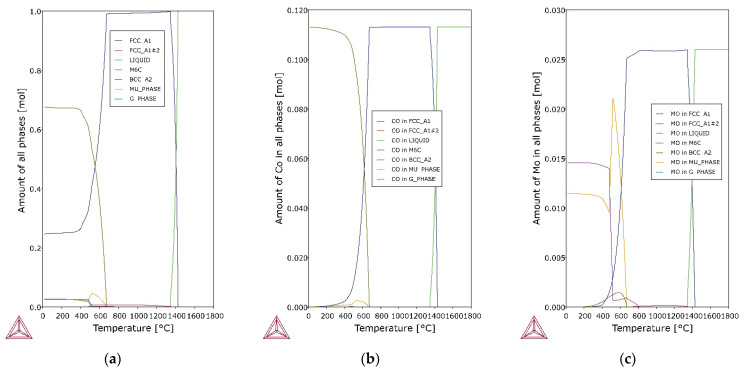
Thermodynamic diagrams of Fe-0.0768C-0.921Si-0.168Mn-4.38Mo-16.9Ni-11.7Co-0.332Ti model alloy: (**a**) amount of all phases, (**b**) amount of Co in all phases, (**c**) amount of Mo in all phases.

In the 6th layer and previously deposited layers, a typical dendritic structure with columnar grains which uniformly distributed layer bands of the martensitic structure and fine dispersion of austenite (white pools) between the dendrites are observed (Figure 3c,d and Figure 4c,d). The absence of austenite in the subsequent layers (7th–10th) suggests that the white pools in the 6th and earlier layers are reverted austenite [68], transformed during reheating from the hardened state. Doubts may be raised by the non-smooth austenite/martensite interface, indicating residual austenite observed e.g. in structural steels [69], but Zhang et al. [70] explained the invasive behaviour of γ grains in such cases. The presence of austenite in the 1st layer (out of 4) of the maraging steel arc weld metal was found by Gupta et al. [71], similar to Tariqu et al. [61]. To assess the austenite content, the 6th layer microstructures were subjected to image analysis. Each of the 5 photos was binarized and then the proportion of black and white zones was measured. Image analysis showed that the share of the austenite fraction share is 11.435 ± 1.313%. Thermodynamic analyses show that at room temperature under equilibrium conditions the share of austenite in the Fe-0.0768C-0.921Si-0.168Mn-4.38Mo-16.9Ni-11.7Co-0.332Ti alloy is approximately 25% (Figure 5a). The difference between the values is due to the non-equilibrium conditions during WAAM.

The hardness of 18Ni-12Co-4Mo-Ti steel is not uniform on the cross-section. The material is harder in the 6th and subsequent layers (525 ± 11 HV10) than in the 10th–8th layers (375 ± 12 HV10). These results suggest that the last layers are in a supersaturated state, while the previous layers, due to successive transient thermal cycles of manufacturing, are uncontrolled age hardened.

**Figure 6:**
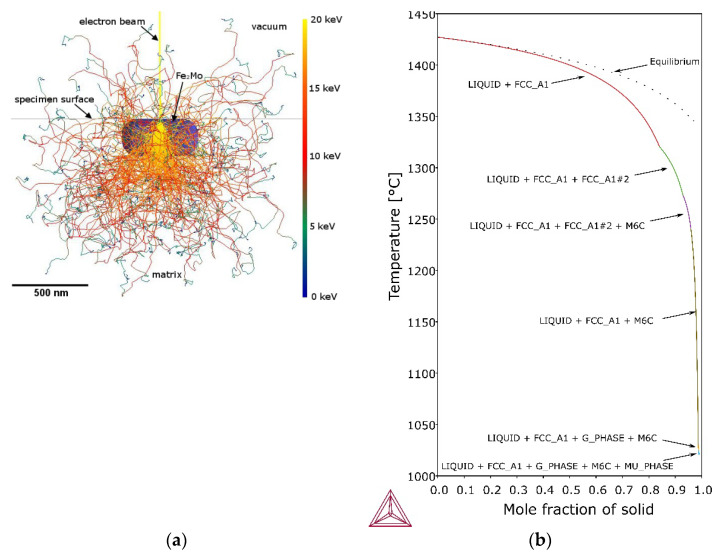
Monte Carlo simulation of electron trajectory in Fe2Mo precipitate and Fe-0.0768C-0.921Si-0.168Mn-4.38Mo-16.9Ni-11.7Co-0.332Ti model alloy matrix (**a**) and Scheil solidification simulation of Fe-0.0768C-0.921Si-0.168Mn-4.38Mo-16.9Ni-11.7Co-0.332Ti model alloy matrix (**b**).

Analysis of the results of EDS analysis, the crystallization process and thermodynamic calculations allows for more precise conclusions about the microstructure and primary precipitates in maraging steel after WAAM. In the 10th layer, the primary precipitates rich in titanium and zirconium (point A in Figure 4b), as well as molybdenum and titanium (points B, C and D in Figure 4b), are observed. They are located mainly in intercellular spaces, where there is also segregation of elements with a low solute partition coefficient. As noted by Sha et al. [72] intermetallic compounds are formed during primary crystallisation without eutectic transformation, because of which they distribute much more evenly than even carbides. Primary precipitations form during solidification in the interdendritic areas, which are generally rich in alloying elements. This phenomenon is observed both in steels [73] and nickel [74] or aluminum [75,76,77] alloys. It is also emphasized that the intermetallic particle size is usually smaller, up to 2–3 μm in diameter [72], which corresponds to the observation (Figure 4). The relatively small size of the precipitates is responsible for the high iron, nickel and cobalt content, which could come from the matrix. An example of the effective depth of electron penetration in the chemical analysis of an exemplary small-size Laves phase Fe_2_Mo precipitate, performed by Monte Carlo Simulation, is presented in Figure 6a. Characteristic X-rays were emitted approximately equally from the inclusion and the matrix. Scheil simulation (Figure 6b) shows that austenite crystallizes from the liquid, and then M_6_C carbides, G-phase and μ-phase intermetallic compounds are precipitated, to reach 100% of the solid at a temperature of about 1020 °C. Equilibrium thermodynamic analysis shows that only a small amount of cobalt is part of the μ-phase in the range 500–650 °C (Figure 5b), and with the γ → α transformation progress, the rest is in martensite. The high molybdenum content in the primary precipitates B, C and D indicates μ-phase (Figure 5c), described by the formula (Fe,Mn,Ni,Co)_7_Mo_2_(Fe,Mo,Ni,Co)_4_. The increased titanium content may come from the matrix, enriched with this element in the intercellular spaces. Zirconium, which was not identified in the OES analysis (Table 3) due to its low volume fraction, was found locally in the precipitates. Its origin may be related to the use of this element as a deoxidizer in the flux-cored wire.

Similarly, primary precipitations are located in the 6th layer and additionally, austenite is visible in the intercellular spaces. Due to the segregation of elements during crystallization, austenite (point J, Figure 4d) is rich in Ti (1.04%) and Ni (19.01%) compared to martensite (point K, Figure 4d) where is 0.16%Ti and 16.34%Ni. No significant difference in cobalt content. There is no significant difference in the chemical composition between the precipitates of B, F and G, which may indicate that the thermal cycles of successive layers do not significantly affect the chemical composition of the primary precipitates. The lower nickel content in points F and G compared to B may indicate an increase in the nickel content in the matrix around the precipitation. This enriches the matrix locally with nickel and encourages the formation of austenite. The nickel content (point J, Figure 4d) is significantly higher than in the martensitic matrix (point K, Figure 4d).

Locally, silicon-rich cubic precipitates were also observed (point E, Figure 4d), with a chemical composition corresponding to precipitates (Fe,Co,Ni,Mo)_2_Si_5_, formed in the Fe-Si system in the range of 67–73.5% at. Si. Similarly, aluminium-rich precipitations are observed locally (point H, Figure 4d), with the chemical composition corresponding to the precipitates (Fe,Co,Ni,Mo,Ti)_3_Al, released in the Fe-Al system with α’ at 550 °C.

For a deeper understanding of the microstructure features, diffraction and TEM studies seem appropriate. An individual experiment is required to confirm the austenite transformation during WAAM. The obtained results indicate, however, that in the present state the product is not the best input material for subsequent production processes. The occurrence of primary precipitation of intermetallic compounds causing the depletion of the matrix with alloying elements will consequently result in probably less hardening of the alloy after the aging heat treatment. Also, the anisotropy of the structure and properties on the cross-section, especially including the last four layers of the product, can have a significant impact on the characteristics of the finished product.

## 4. Conclusions

In the presented work, the part made of 18Ni-12Co-4Mo-Ti maraging steel was prepared and characterised with the use of optical emission spectrometry, light microscopy and scanning electron microscopy with energy dispersive spectroscopy. It is possible to produce a maraging steel part using the WAAM process with flux-cored wire. The use of flux-cored wire made enabled to obtain relatively wide and shallow layers with a regular circular shape. Although the individual layers are covered with a thin layer of slag, no internal defects are observed. The collinearity of individual layers has not been maintained, which may be associated with the unstable straightening of the wire after it slips out of the torch current tip. The addition of COREWELD NiCoMo flux-cored wire resulted in the chemical composition of the part being similar to 1.6356 maraging steel. Wire arc additive manufacturing heat cycles led to a clear division of the part into two areas. The last layers with a hardness of 375 ± 12 HV10 were characterized by a supersaturated martensitic microstructure with primary precipitates of intermetallic compounds, located mainly in intercellular spaces. The further layers with a hardness of 525 ± 11 HV10 were characterised by an aged martensitic microstructure. Furthermore, white pools of austenite, whose area share was 11.435 ± 1.313%, were observed in the intercellular spaces, most likely due to reverse transformation. Heat cycles did not significantly affect the primary precipitates of intermetallic compounds, which likely the μ-phase.

## Figures and Tables

**Table 1:** Wire arc additive manufacturing process parameters.

Layer	Mean Current I (A)	Mean Voltage U (V)	Wire Feed Speed v_w_ (m/min)	Manufacturing Speed v_m_ (m/min)	Electro-Stick Out (mm)
1–10	143.5	18.7	4.25	0.375	12

**Table 2:** Chemical composition of tested 18Ni-12Co-4Mo-Ti maraging steel. Mass %.

Element	C	Mn	Si	Ni	Co	Mo	Ti
WAAM product (OES)	0.0768	0.168	0.921	16.9	11.7	4.38	0.332
COREWELD NiCoMo (manufacturer data)	0.03	−	0.3	18.0	12.0	4.0	+
Werkstoffnummer 1.6356 (DIN standard)	≤0.03	≤0.1	≤0.1	17.0–18.5	11.5–13.5	3.0–4.5	1.5–2.0

**Table 3:** Average values of elements content in EDS analysis for points A–K shown in Figure 4. The rest are Fe and C.

Element	Point A	Point B	Point C	Point D	Point E	Point F	Point G	Point H	Point J	Point K
Mn	0.38	0.37	0.19	0.18	-	0.17	-	0.15	0.29	-
Si	1.71	5.60	1.74	4.01	61.25	6.38	6.33	0.57	1.88	1.71
Ni	8.46	13.74	10.61	14.06	4.90	12.89	12.95	11.78	19.01	16.34
Co	7.20	10.66	8.95	10.91	6.57	10.48	10.90	12.23	13.66	13.70
Mo	5.37	29.92	32.30	24.67	1.13	33.88	34.10	2.71	7.44	4.07
Ti	5.87	3.70	6.59	5.64	-	3.37	3.30	0.91	1.04	0.16
Zr	39.29	2.71	1.62	1.76	-	-	-	-	-	-
Al	-	-	-	-	-	-	-	18.66	-	-

## Data Availability

The data presented in this study are available in article.
